# The Right ventricle and cardiac surgery - more resilient than thought: multiparametric quantification shows altered rather than reduced function

**DOI:** 10.1186/1532-429X-18-S1-P140

**Published:** 2016-01-27

**Authors:** Katia D Menacho Medina, Thomas A Treibel, Rebecca Kozor, Heerajnarain Bulluck, James Moon, Charlotte Manisty

**Affiliations:** Barts Image Center, Bartholomew Hospital, London, UK

## Background

Right ventricular (RV) function appears to declines following cardiac surgery, particularly long axis function. Three principal speculated causes are suggested: a) loss of constraint when pericardium is opened, b) intra-operative ischemia, c) post-operative tethering. The relative contributions are unknown. Similarly, the impact on different aspects of RV function (AV plane descent, apical movement towards the AV plane) is obscure.

The length and complexity of cardiac operations is also thought to contribute to RV impairment. We sought to untangle these relative contributions by comparing pre- and post-surgery RV function changes in a cohort of patients where all had aortic valve replacement (AVR) but 1/3 had in addition grafting (CABG). Different measures of RV function were used and paired pre and post.

## Methods

**Figure 1:**
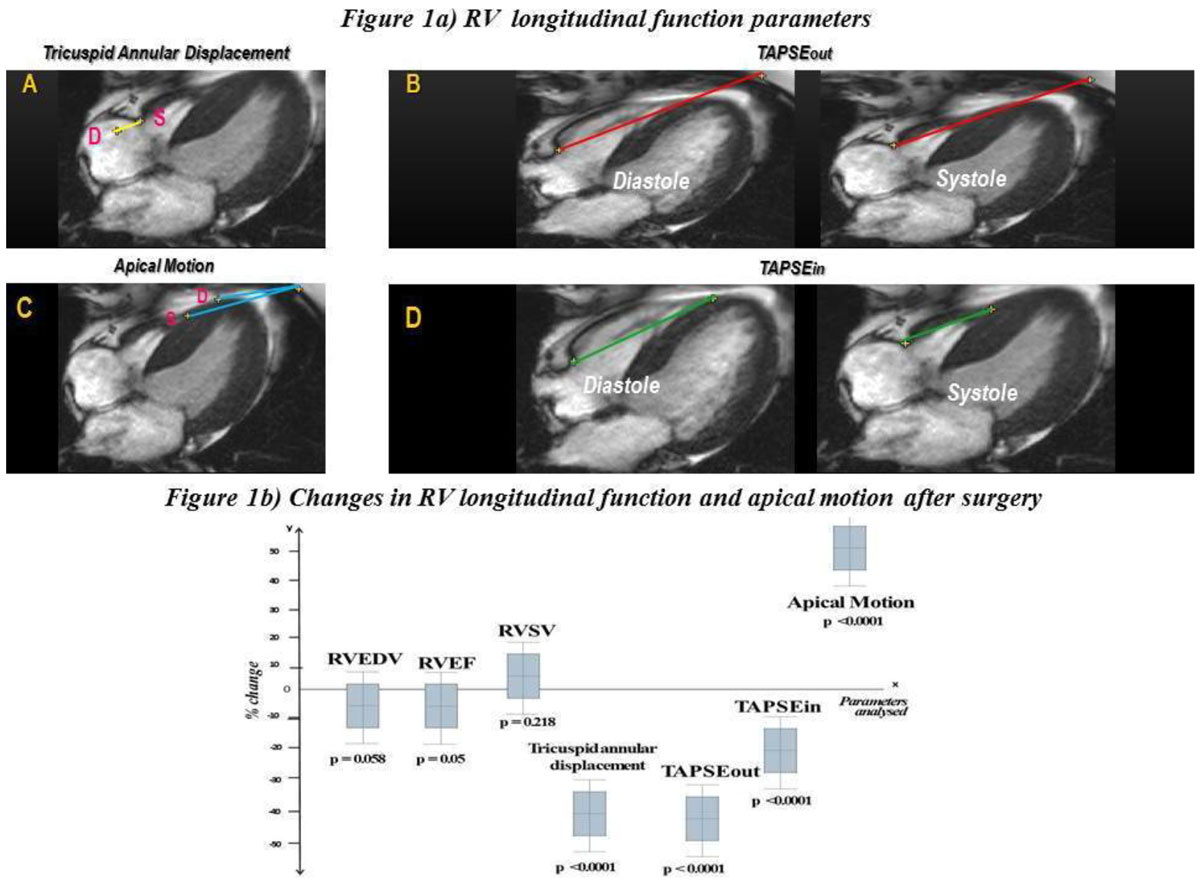
**Right ventricular longitudinal function assessment and changes post-surgery**.

Eighty-one patients were scanned at 1.5T (Siemens Avanto) prior to, and 1 year following AVR (n = 59) or combined AVR and CABG (n = 22), with full pericardial opening. RV volumes and function were calculated using standard techniques. RV longitudinal function was assessed using different measures, Figure 1(a).

A - Tricuspid annular displacement - the distance measured between tricuspid annulus in diastole and systole

B - TAPSE_out_ - the difference in the distance between tricuspid annulus and a fixed point on anterior chest wall in diastole and systole

C - RV apical motion - the difference in the distance between the RV apex and a fixed point on the anterior chest wall in systole and diastole.

D- TAPSE_in_ - the difference in the distance between tricuspid annulus and RV apex in diastole and systole (incorporates apical motion)

## Results

RV volumes and ejection fraction were within normal limits for all subjects, pre and post, with no significant reductions in RV function and volumes, Table 1.

**Table 1:** Changes on right ventricular function and apical motion after surgery

	Pre-surgery mean value	Post-surgery mean value	p value
RVED (ml)	111 ± 33	115 ± 31	0.058
RVEF (%)	68 ± 6	65 ± 9	0.05
RVSV (ml)	75 ± 14	78 ± 13	0.218
Tricuspid annular displacement	21 ± 4	12 ± 4	<0.005
TAPSEout (mm)	19 ± 4	11 ± 3	<0.001
RV apical motion (mm)	5 ± 3.7	7.7 ± 4.9	<0.001
TAPSEin (mm)	24 ± 7	19 ± 5	<0.001

RV longitudinal function however assessed using tricuspid annular displacement and TAPSE_out_ declined after surgery by 41% (*p < 0.0001*) and 43% (*p < 0.0001*), respectively. Apical motion increased post operatively by 52% (*p < 0.0001*), meaning that overall TAPSE_in_ only fell by 21% (*p < 0.0001*), Figure 1(b).

RVEF had weak correlation with each of the markers of RV longitudinal function, (r = 0.30 for tricuspid annular displacement, *p < 0.01*). Using tricuspid annular displacement <16 mm alone, 68 out of 81 patients (84%) would be classified as having impaired RV function post-operatively, despite preserved RVEF.

There were no significant differences between the AVR+CABG and AVR alone groups in any of the measures of RV function (RVEF: 65 ± 9% versus 64% ± 9%, *p = 0.88*) respectively.

## Conclusions

The effect of cardiac surgery on overall RV function is overstated - yes, tricuspid annular displacement falls, but apical basal contraction increased in compensation.

Doing additional grafts has no detectable incremental detrimental effect on RV function.

Ischemia does not appear a likely cause of changes - tethering and/or loss of pericardial constraint appear to be the principal mechanisms of RV changes.

